# Editors’ Book Table

**Published:** 1875-03

**Authors:** 


### Editors' Book Table.

[NOTE. — All works reviewed in the pages of the CHICAGO MEDICAL JOURNAL may be found in the extensive stock of W. B. KEEN, COOKE & Co., whose catalogue of Medical Books will be sent to any address upon request.]

MATERIA MEDICA, FOR THE USE OF STUDENTS. By *John B. Biddle*, Professor of Materia Medica and General Therapeutics in the Jefferson Medical College, etc., etc. Sixth Edition, revised and enlarged, with Illustrations. Philadelphia: Lindsay & Blakiston. 1874. $4.00.

In this modest little volume of four hundred pages we have the best compendium of Materia Medica in the language, whether considered with reference to comprehension or to its method. The subject matter comprises all that is necessary for the student to acquire, and very much more than one in a thousand practitioners ever learns, while its arrangement is systematic, concise and simple.

THE OUTLINES OF THE SCIENCE AND PRACTICE OF MEDICINE. By *William Aitken, M.D., F.R.S.*, Professor of Pathology, in the Army Medical School, etc., etc., London: Chas. Griffin & Co., 10 Stationers' Hall Court. Philadelphia: J. B. Lippencott & Co. 1874. $5.00.

The medical student of the present is peculiarly fortunate in the greatly increased facilities for the acquisition of professional knowledge which he enjoys, as compared to those of his predecessor of a few years ago. Amongst the many books "designed for the use of students," it would be hard to find one as comprehensive and unobjectionable as this. It is not a "Compendium" or a "Vade Mecum," and differs essentially from the whole of that class, but is really what it pretends to be, "The Outlines of the Science and Practice of Medicine," containing what is essential for the student to know, and omitting much that is superfluous.

We will, however, venture to object to the propositions contained in the first chapter, *i. e.*, that on "preliminary definitions and explanations," viz: "Health implies that state or condition in which a person exists, being fully able, without suffering, to perform all the duties and functions of life, possessing the normal activity of all the component parts of the body. Thus many degrees of health are possible, from the possession of a feeble existence to the most robust constitution." The author desires, doubtless, to express the fact that many degrees of vigor are compatible with the idea of health in different individuals, although his mode of expression is somewhat paradoxical, for it is difficult to imagine one "possessing but a feeble existence," and yet "performing all the functions and duties of life," unless very imperfectly.

The next proposition, *i.e.*, "The phenomena of disease are the *normal* vital manifestations of the body under the influence of some unusual, hurtful or dangerous condition, which is known as the 'morbific cause,'" appears to involve a verbal contradiction. "Normal vital manifestations," under the operation of "morbific causes," must have already lost their *normal* and assumed an abnormal character in order to appear as "phenomena of disease."

The next proposition in its first term is too exclusive, *i. e.*, "Disease is therefore some deviation from this normal activity of function;" and does not comprehend even the simplest manifestations of disease. The next term suggests as a possible, a necessary condition, *i. e.*, "If the deviation from the normal activity of function be accompanied by appreciable alteration of structure, the disease is then said to be structural or organic,"—and is moreover indeterminate in its phraseology, as the word "appreciable" is purely relative, and may refer to every degree of perception, from the unaided observation of a tyro to the trained eye of the expert microscopist with his 1-50 inch objectives.

The third term of this proposition involves a conclusion which seems utterly unwarranted by observation of fact, or by analogy, *i. e.*, "If there be no appreciable change of structure, the disease is said to be functional." No objection can be made to the form of the argument, but to the premises which involve a petitio principii. "Functional disease" has a strange old fashioned sound at this day, when Beale and Frey and Richardson are demonstrating daily the precedence of structural over functional derangements, and illustrating with their high power lenses, what was already so clear to the logical mind, that function presupposed structure, and could not be manifested either in its normal or its morbid phases, without corresponding *pre-existing* structural conditions.

True scientific progress is always from the complex to the simple, from the accumulated data to the general principle underlying them, in view of which we have endeavored to indicate what we consider a defect in a book so full of merit and value, which could not fail to impair its usefulness to the student by the inculcation of principles which scientific progress has long since shown to be only acceptable while awaiting the light that has long since dawned upon us. H.

FREE PHOSPHORUS IN MEDICINE, WITH SPECIAL REFERENCE TO ITS USE IN NEURALGIA: A Contribution to Materia Medica and Therapeutics. By *J. Ashburton Thompson*, Surgeon at King's Cross to the Great Northern Railway Company, Surgeon Accoucheur to the Royal Maternity Charity. London: H. K. Lewis, 136 Gower Street, W. C. 1874.

This work is modestly announced by the author in his preface as "Notes," "not so much constituting an original work, as containing a resume of what is known of the use of phosphorus in medicine, written in the light of considerable clinical experience."

It comprises the history, pharmaceutical preparation, dose, internal administration, therapeutic uses, cases of poisoning, and biography.

The first chapter is purely historical, and of interest only in that regard. The second chapter, relating to the pharmaceutical preparations of the drug, has a practical interest. The solutions of phosphorus in the various oils are discussed in detail, and do not receive the author's approbation, in view of the chemical reactions established, through which "the activity of the dissolved element" may be "seriously altered, impaired or destroyed." Of these oily solutions he gives the preference, as would be expected, to that in cod liver oil.

Regarding the solutions in ethers, alcohol and the hydro-carbons, the exercise of extreme care is enjoined to secure an absolutely anhydrous condition in the solvent, as "the presence of a single drop of water is sufficient to throw down some of the element in its hydrated form and thus cast doubt on the actual strength of the solution."

Of these solutions, the tincture of phosphorus, prepared by boiling an excess of the drug in absolute alcohol, and diluted, for administration, with alcohol, glycerine and aromatics, in such proportions that one-twelfth of a grain shall constitute a dose, appears to meet the author's approval, while the ethereal solution he rejects, as nauseous, and that with chloroform, on account of the therapeutic uncertainty of the solvent. Of aqueous solutions he speaks thus: "Phosphorized water, however, is obviously a preparation too unsalable and too uncertain in its composition to be employed in medicine." The use of the drug in solution, in capsules, is objected to on account of the solvent usually employed and the necessary minuteness of the dose; and to its use in pill form many valid objections are urged, such as the difficulty of effecting minute subdivision, liability to ignition, inequality of doses, and tendency to decomposition.

The author's investigations have led him to reject as inert, so far, at least, as any of the effects of free phosphorus are considered, the hypophosphites of sodium and calcium—and this conclusion we most heartily endorse whether these preparations be considered in regard to the therapeutic effect of phosphorus, or of anything else, which they may be supposed to contain.

The phosphide of zinc appears to the author to possess greater advantages and fewer disadvantages for therapeutic purposes than any other preparation of the drug, and this conclusion seems to be sustained by the collateral testimony of contemporary observers. Allotropic, or red phosphorus, is regarded as inert except in so far as it may contain free phosphorus, from which it may be separated by careful washing with bisulphide of carbon.

Regarding zinc phosphide, and other forms, the author formulates the following propositions:

1st. "That solutions of phosphorus in virgin vegetable oils are not safe, and should therefore be entirely rejected."

2nd. "That the solid form is not a perfectly safe mode of administering phosphorus; it may, however, be employed, but should never be presented to the empty stomach."

3rd. "That the administration of the zinc phosphide should be attended by the use of an acid at the same time."

Of the first forms he suggests no dose. In solution in cod liver oil he suggests the equivalent of one-twelfth of a grain, to be reduced, after continued use during ten days. As a tonic, one-fiftieth of a grain is directed. Of the tinct. phosphide the full dose is said to be three-quarters of a grain, and an average one-third of a grain every two, or, better, every four hours.

In comparison with the dose of other preparations of the drug, the doses of the zinc phosphide here suggested seem disproportionately large, much larger than the dose of the same preparation recommended by Dr. Hammond, the first American authority who has experimented with it; and in view of the rapid supervention of poisonous effects, and their unmanageable character, a note of caution should be appended to this suggestion of the use of such large doses.

Regarding the general therapeutic action of phosphorus, the author's observations confirm those of preceding observers; the cumulative effects noticed by some, he has failed to observe.

The special point of interest in the book, however, is the curative value claimed for the drug, in several forms of nervous disease, but more especially in neuralgias, for the treatment of which, the author regards it almost in the light of a specific, supporting his arguments by reports of many cases, of which he has tabulated fifty, the larger proportion of which recovered or improved under its influence.

While a large experience in the management of this form of nervous disease, and a thorough appreciation of its refractory nature under any mode of treatment, will not permit us to accept, without a doubt, all the author's deductions, it is reasonably certain that we must look to phosphorus as one of the most valuable agents at our command for the improvement of nerve nutrition and the removal of morbid conditions resulting from the perversion of that process.

In any event, Dr. Thompson is entitled to and should receive the thanks of the profession for this really valuable contribution to the literature of neuro-therapeutics, both for the matter treated and the manner of its treatment, a book which will prove well worth the time spent in its perusal by any one, and which no neurologist can afford to neglect. H.

Nomenclature of Disease. Prepared for the Use of the Medical Officers of the United States Marine Hospital Service, by the Supervising Surgeon (*Jno. M. Woodworth, M.D.*); being the classification and English Latin Terminology of the Provisional Nomenclature of the Royal College of Physicians, London. Washington: Government Printing Office. 1874.

Dr. Woodworth has hereby added another to the many improvements instituted by him in the administration of the Marine Hospital service since its reorganization, or rather its organization, for before his advent it could scarcely be said to have had a very definite organization. The classification of statistics is the first step toward their utilization, and this classification is, frequently, indeed generally, embarrassed or altogether prevented by the absence of uniformity in nomenclature. The work before us has been accomplished to remedy this difficulty, and will permit the classification of the large mass of statistics, susceptible of accumulation by this department of the Civil Service of our government, which have hitherto been lost to science.

The adoption of the Provisional Nomenclature of the Royal College of Physicians has some advantages, for while it is by no means perfect, nor even the best that might have been devised, it is still recognized as the key to so large a mass of statistics, and what it lacks in accuracy, is more than supplemented by its uniformity and comprehension.

Dr. Woodworth is entitled to great credit for the efficient manner in which he has administered the affairs of his Department, of which, perhaps, one of the best criteria is the lack of appreciation manifested in Congress. It does n't pay. H.

CROUP IN ITS RELATIONS TO TRACHEOTOMY. By *J. Solis Cohen, M.D.*, Lecturer on Laryngoscopy and Diseases of the Throat and Chest, in Jefferson Medical College. Philadelphia: Lindsay & Blakiston. 1874.

The above is a typical example of a most valuable class of works for which general practitioners can never be too grateful to authors, who in these records of their own labors and observations, collated with those of others, thoroughly exhaust the subject, and present its merit in a condensed form to their readers, who are thereby saved days and nights of, oftentimes, fruitless toil in the search after "the grain of wheat in the bushel of chaff."

Dr. Cohen's conclusions concerning the use of tracheotomy in croup are summarized thus:

1. That there are no insuperable contra-indications to tracheotomy in croup;

2. That the administration of an anæsthetic for the purpose of controlling the child's movements is admissible in performing the operation; but that it should be used with great caution;

3. That a careful dissection should be made down to the windpipe, and hæmorrhage be arrested before incising it, whenever there is at all time to do so;

4. That the incision should be made into the trachea as near the cricoid cartilage as possible, to avoid excessive hæmorrhage, and subsequent accidents which might occasion emphysema;

5. That a dilator should be used, or a piece of the trachea be excised, whenever any difficulty is encountered in introducing the tube;

6. That the tube should be dispensed with as soon as possible; or altogether, if the case will admit of it;

7. That assiduous attention should be bestowed upon the after treatment, especially that of the wound; and that a skilled attendant should be within a moment's call for the first twenty-four or forty-eight hours immediately following the operation."

The justice of one of the author's initial propositions cannot fail to be appreciated by every conservative mind, *i.e.*: "Tracheotomy, in itself, does not cure croup. It affords a possibility of recovery by postponing, or insures it by averting, death. The course of the disease is continued until all its attendant phenomena have undergone evolution. The surgeon's knife merely cuts a path for the air to reach the bronchi in quantity sufficient to meet the requirements of the respiratory process, and saves the muscular force exhausted in futile efforts at respiration through the glottis."

Dr. Cohen's little book will become one of the classics. No one who has ever seen a case of croup will fail to derive both pleasure and profit in reading it, and no one who ever expects to see a case can afford to deprive himself of the knowledge which he will thereby acquire.

THE BREATH, AND THE DISEASES WHICH GIVE IT A FETID ODOR: with Directions for Treatment. By *Joseph W. Howe, M.D.*, author of "Emergencies," Clinical Professor of Surgery, in the Medical Department of the University of New York; Visiting Surgeon to Charity Hospital; Fellow of the New York Academy of Medicine, etc. New York: D. Appleton & Company. 1875.

It is somewhat remarkable that the subject of fetid breath, which occasions so much annoyance and even mental suffering to its victims, and disgust to all who come within the range of its influence, should have attracted so little attention from authors and investigators. Hence, a thoroughly scientific exposition of the whole subject, such as Dr. Howe has given us, has long been a desideratum.

The contents of the little volume before us comprise "the physiology of repair, decay and respiration, fetid odors from emotion, from dyspepsia, from bad teeth and ulcers of the mouth, from catarrhal disorders, and from mineral poisons; the whole contained in a little volume of one hundred pages, which well deserve the attention of physicians, to whom we commend it most highly.

H.

THE DISEASES OF THE STOMACH; Being the third edition of the "Diagnosis and Treatment of the Varieties of Dyspepsia." Revised and Enlarged by *Wilson Fox, M.D., F.R.C.P., F.R.S.*, Physician Extraordinary to Her Majesty the Queen; Fellow of University College; Corresponding Member of the Psyikalisch-Medicinisch-Gesellschaft of Wurzburg; Holme Professor of Clinical Medicine, University College, London; Physician to University College Hospital. With illustrations. Philadelphia: Henry C. Lea. 1875.

An excellent monograph concerning a class of diseases from which few of us frail mortals are exempt at some time or other in our lives, is presented in the above-named treatise. The author wisely takes for his point of departure, the proposition that pathological conditions are only disturbances of physiological order, and that no new factors are introduced.

Proceeding from such a basis, it is scarcely possible to wander far from the truth,—within the limits of what is clearly defined in physiology. The book consists of two divisions, of which the first comprises the symptomatology of the stomach, including the various appearances of the tongue, derangements of the appetite, thirst, flatulence, acidity and pyrosis, pain, vomiting, indigestion. Of these, the last four subjects are discussed admirably, and the chapters assigned to them are especially worthy of study, as clear and succinct statements of the true pathology of the morbid conditions which express themselves by these symptoms, as deduced from the most recent physiological data.

The student and general practitioner who is not perfectly sure of his physiology cannot devote a few hours to a better purpose than by a careful, thoughtful study of the subject as formulated by this author in these few chapters.

The second division of the book, comprising the special pathology of the stomach, comprehends the subjects, atomic dyspepsia, nervous, acute and chronic catarrh, ulcer, cancer, hæmorrhage, hypertrophy, stricture of the cardiac orifice, obstruction of the pylorus, dilatation, softening, perforation, rupture, tubercle. All of which subjects are discussed in a manner which cannot fail to be both agreeable and instructive to the reader. The subject of the neuroses of the stomach, receives much more attention than is usually assigned to it by systematic writers, a degree of attention which it well deserves, and, indeed imperatively demands, in view of the very indefinite ideas which are entertained just now by so many physicians, of the correlation of nervous functions, and those—so-called, more especially—of organic life. The therapeusis here taught is rational, and as free from the spirit of empiricism as can reasonably be expected, when the crude state of therapeutics is considered.

Amidst all its excellencies, the book is not altogether free from defects, although they are for the most part trivial ; it is to be regretted, however, that the author, or his proof reader, should have sacrificed his diphthongs on the altar of the demon of willful ignorance, and thereby contributed to the progress of phonetic corruption which the English language is doomed, like all of its predecessors, to suffer. *Hemorrhage* has no meaning, unless one which is purely arbitrary, for it has no derivation, no philological relations ; and the same may be said of Etiology. These are minor defects, but they nevertheless detract from the general excellence of the book, and from the scholastic merit of the author.

### BOOKS RECEIVED.

A Practical Treatise on the Medical and Surgical Uses of Electricity, including Localized and General Faradization, Localized and Central Galvanization, Electrolysis and Galvano-Cautery. By *George M. Beard, A.M., M.D.*, Member of the New York Society of Neurology and Electrology, etc., etc.; and *A. D. Rockwell, A.M., M.D.*, member of the New York Society of Neurology and Electrology, etc., etc. Second Edition, revised, enlarged and mostly re-written, with nearly two hundred illustrations. New York: William Wood & Co. 1875.

Dental Pathology and Surgery. By *S. James Salter, M.B., F.R.S.*, member of the Royal College of Surgeons, etc., etc. New York: William Wood & Co. 1875.

On the Treatment of Pleurisy; with an appendix of cases, Showing the Value of Combination of Croton Oil, Ether and Iodine, as Counter Irritants in other Diseases. By *Jno. W. Carson, M.D.*, late Physician to the class of Diseases of the Chest, in the N. Y. and Eastern Dispensaries, etc. N. Y.: W. Wood & Co. 1874.

Compendium of Children's Diseases: a Handbook for Practitioners and Students. *By Dr. Johann Steiner*, Professor of the Diseases of Children in the University of Prague, and Physician to the Francis-Joseph Hospital for Sick Children.

Translated from the Second German Edition by Lawson Tait, F.R.C.S., Surgeon to the Birmingham Hospital for Women, Consulting Surgeon to the West Bromwich Hospital, Lecturer on Physiology at the Midland Institute. New York: D. Appleton & Company. 1875.

Physician's Office Case Record Book. 1875. Case Record Company, 224 Laurel st., Cincinnati, Ohio.

Physician's Pocket Case Record Book. 1875. Case Record Company, 224 Laurel St., Cincinnati, O.

### PAMPHLETS RECEIVED.

Ozone, the Active or Electro-Negative Oxygen, its Curative Virtues in Therapeutics. By *Dr. Theo. A. Hoffmann.* Beardstown, Ill. 1875.

Sellers et al. *vs.* The Pennsylvania Railroad Company et al. Complainants' Affidavits on Motion for Injunction to restrain Erection of Slaughter House. In the Court of Common Pleas, of Philadelphia County. In Equity. Dec. Term, 1874. No. 3.

Near Sight, Treated by Atropia; with Tables. By *Hasket Derby, M.D.*, Surgeon to the Massachusetts Charitable Eye and Ear Infirmary, at Boston, etc., etc. New York. 1875.

Migrants and Sailors, Considered in their Relation to Public Health. *A.* Some Defects in the Immigration Service affecting the Sanitary Interests of the Country. By *Jno. M. Woodworth, M.D.*, Supervising Surgeon of Marine Hospital Service. *B.* Sailors as Propagators of Disease. By *Heber Smith, M.D.*, Surgeon U. S. Marine Hospital Service. Cambridge: Printed at the Riverside Press. 1875.

Fourteenth Biennial Report of the Trustees, Superintendent and Treasurer of the Illinois State Hospital for the Insane, at Jacksonville. Dec., A. D. 1874.

Transactions of the American Ophthalmological Society. Tenth Annual Meeting, Newport, July, 1874. New York: Wm. Wood & Co. 1874.

Report of one hundred and five Cases of Operation for Cataract.

A Series of American Clinical Lectures. Edited by *E. C. Seguin, M.D.*, Vol. i, No. 1. On Disease of the Hip-Joint. By *Lewis A. Sayre, M.D.*, Prof. of Orthopædic Surgery and Clinical Surgery in Bellevue Hospital Medical College, New York. G. P. Putnam's Sons. 1875.

### JOURNALS RECEIVED.

The American Medical Weekly—Vol. ii, Nos. 5, 6, 7.
The Archives of Dermatology—January.
The American Practitioner—Vol. xi, February.
The Boston Medical and Surgical Journal—Nos. 4, 5, 6.
The Boston Journal of Chemistry—February.
Braithwaite's Retrospect—January.
The Chicago Journal of Nervous and Mental Disease—January.
The Clinic, Cincinnati—Vol. viii, Nos. 5, 6, 7.
The Chicago Teacher.
The Cincinnati Lancet and Observer—Vol. xviii, No. 2.
The Detroit Review of Medicine and Pharmacy—February.
The Dental Cosmos—Vol. xvii, No. 2.
The Eclectic Medical Journal.
The Indiana Journal of Medicine—Vol. i, No. 10.
The Half-Yearly Compendium of Medical Science—Part xv, January, 1875.
The Laboratory, Boston—January.
The Medical Record, New York—Vol. x, Nos. 6, 7.
The Medical Times, Philadelphia—Vol. xxx, Feb. 6, 13.

The Medical and Surgical Reporter, Philadelphia—Vol. xxxii, Nos. 5, 6, 7.
The Medical Examiner, Chicago—February 1, 15.
The Medical News and Library—Vol. xxxiii, No. 386.
The Medical Sciences, Monthly Abstract of—Vol. ii, No. 2.
The Missouri Clinical Record—Vol. i, No. 2.
The Nashville Journal of Medicine and Surgery—February 7.
The New York Medical Journal—Vol. xxi, No. 2.
New Remedies—January, 1875.
The Psychological and Medico-Legal Journal—February.
The Peninsular Journal of Medicine—Vol. xi, No. 2.
El Repertorio Jalisciense, Guadalajara—No. 6, Tomo i, Enero.
The St. Louis Medical and Surgical Journal—Vol. xii, No. 2.
The Virginia Medical Monthly—Vol. i, No. 11.

